# Study of Printable and Biocompatible Alginate–Carbon Hydrogels for Sensor Applications: Mechanical, Electrical, and Cytotoxicity Evaluation

**DOI:** 10.3390/gels11060389

**Published:** 2025-05-26

**Authors:** Laura Mendoza-Cerezo, Jesús M. Rodríguez-Rego, A. Macias-García, Francisco de Asís Iñesta-Vaquera, Alfonso C. Marcos-Romero

**Affiliations:** 1 Departamento de Expresión Gráfica, Escuela de Ingenierías Industriales, Universidad de Extremadura, Avenida de Elvas, s/n, 06006 Badajoz, Spain; lmencer@unex.es (L.M.-C.);; 2 Departamento de Bioquímica y Biología Molecular y Genética, Facultad de Ciencias, Universidad de Extremadura, Avenida de Elvas, s/n, 06006 Badajoz, Spain; 3 Departamento de Ingeniería Mecánica, Energética y de los Materiales, Escuela de Ingenierías Industriales, Universidad de Extremadura, Avenida de Elvas, s/n, 06006 Badajoz, Spain

**Keywords:** conductive hydrogels, carbonaceous materials, 3D printing, biocompatibility, biosensors

## Abstract

The development of printable, conductive, and biocompatible hydrogels has emerged as a promising strategy for the next generation of flexible and soft sensor platforms. In this study, we present a systematic investigation of alginate-based hydrogels incorporating different carbonaceous materials, natural graphite, carbon black (Vulcan V3), and activated carbon (PCO1000C), to evaluate their suitability for sensor applications. Hydrogels were formulated with varying concentrations of sodium alginate and a fixed loading of carbon additives. Each composite was characterized in terms of electrical conductivity under compression, rheological behavior, and mechanical strength. Printability was assessed using a custom-designed extrusion platform that allowed for the precise determination of the minimum force and optimal conditions required to extrude each formulation through a standard 20G nozzle. Among all tested systems, the alginate–graphite hydrogel demonstrated superior extrudability, shear-thinning behavior, and shape fidelity, making it well-suited for 3D printing or direct ink writing. A simple conductivity-testing device was developed to verify the electrical response of each hydrogel in the hydrated state. The effects of different drying methods on the final conductivity were also analyzed, showing that oven drying at 50 °C yielded the highest restoration of conductive pathways. Mechanical tests on printed structures confirmed their ability to maintain shape and resist compressive forces. Finally, the biocompatibility of the printed alginate–graphite hydrogel was validated using a standard cytotoxicity assay. The results demonstrated high cell viability, confirming the material’s potential for use in biomedical sensing environments. This work offers a robust framework for the development of sustainable, printable, and biocompatible conductive hydrogels. The combined performance in printability, mechanical integrity, electrical conductivity, and cytocompatibility highlights their promise for flexible biosensors and wearable sensor technologies.

## 1. Introduction

Polymer hydrogels are highly hydrated three-dimensional networks that have attracted increasing interest due to their unique combination of flexibility, biocompatibility, and water absorption [1].

These properties make them promising candidates for a wide variety of advanced applications, including tissue engineering, controlled drug release and, more recently, sensor development. In particular, conductive hydrogels, because of their excellent mechanical and conductivity properties, are especially promising for portable strain sensors and for the configuration of versatile platforms for signal transduction such as in medical diagnostics or health monitoring [2,3].

Among these functional materials, carbon compounds such as activated carbon (PCO1000C; high surface area and micro/macroporosity), carbon black (Vulcan V3; spherical particles and high porosity), and graphite (lamellar crystalline structure) are widely used because of their high electrical conductivity, large specific surface area, and excellent chemical stability [4,5,6]. This selection covers a representative range of electrical conductivity and textural properties. Graphite, with its ordered structure, offers the highest electrical conductivity [7], while Vulcan V3 offers a combination of good dispersibility and moderate conductivity [8]. The comparative analysis of these three phases herein was aimed at identifying the most suitable material for integration into printable functional hydrogels for sensory purposes.

The incorporation of these materials into a hydrogel matrix makes it possible to obtain conductive compounds suitable for electrochemical or pressure sensor applications [9,10]. The synergy between the conductive network provided by the carbonaceous phase and the ionic permeability of the hydrogel favours efficient signal transfer and a sensitive response to external stimuli [11].

However, achieving homogeneous dispersion and stable integration of the carbonaceous particles within a hydrogel matrix remains a technical challenge. Furthermore, for practical implementation in sensor devices, the formulated materials must also exhibit adequate mechanical properties, good reproducibility, and compatibility with printing techniques.

In this context, sodium alginate was selected as the polymer matrix because of its high biocompatibility, ability to form hydrogels at room temperature through ionic crosslinking with calcium [12,13,14,15], and wide commercial availability at low cost. Unlike other polymers used in bioprinting, such as methacrylated gelatin (GelMA) or polyethylene glycol (PEG), alginate does not require prior chemical reactions or photopolymerization, which simplifies its preparation and reduces the risk of cytotoxicity associated with toxic crosslinking agents such as methacrylic anhydride [16], maleimide [17] or glutaraldehyde [18]. Furthermore, its pseudoplastic behavior [19] and its ability to maintain printed structures with high fidelity [20] make it a suitable choice for additive manufacturing applications.

This work aims to develop and characterize printable conductive hydrogels based on alginate and different carbonaceous materials. A comparative analysis of their electrical, mechanical and rheological properties was carried out. In addition, the ability of the mixture to be used in a 3D printing system and its biocompatibility were validated by means of cytotoxicity tests, which reinforced its applicability as a functional platform in the design of sensors, especially in the biomedical field and in portable devices.

## 2. Results and Discussion

This section presents the methodology and results of the development and characterization of alginate-based conductive hydrogels incorporating different carbonaceous materials. The aim was to evaluate their suitability for printing and sensing applications through a comparative analysis of drying behavior, electrical conductivity, rheological response, and extrusion performance.

The selected carbon additives, PCO1000C (activated carbon), Vulcan V3 (carbon black), and natural graphite, were chosen for their high conductivity, surface area, and chemical stability [21]. Sodium alginate was used as the hydrogel matrix due to its biocompatibility and ionic crosslinking ability, which support structural integrity and tunable mechanical properties [22]. Their combination enables the formulation of printable and stable hydrogels with enhanced functionality for sensor applications [23].

### 2.1. Electrical Characterization of Starting Materials

Electrical characterization allowed understanding the behavior of these materials when exposed to electric fields and evaluating their potential in electrochemical applications. In the specific case of alginate and carbonaceous materials, electrical characterization provided valuable information on their electrical conductivity.

Table 1 shows the electrical conductivity measurements at room temperature of the studied carbonaceous elements and sodium alginate, used in their original form and without undergoing any hydration process and at constant pressure.

The table above shows that the samples had the following decreasing order of electrical conductivity: graphite ˃ Vulcan V3 > PCO1000C ˃ alginate. Graphite, as expected, exhibited the highest electrical conductivity (6.12 (Ω·cm)^−1^). This high conductivity is attributed to the unique arrangement of carbon atoms in its crystalline structure. All other materials showed a significant reduction in electrical conductivity. Specifically, Vulcan V3 exhibited an electrical conductivity of 2.21 (Ω·cm)^−1^. This reduction in conductivity compared with graphite may be due to its porous structure and the presence of impurities [24]. Overall, Table 1 reveals a wide range of electrical conductivities among the materials studied.

PCO1000C and sodium alginate offered the lowest conductivities. Understanding these properties is crucial for the proper selection of materials for various applications. Carbonaceous materials such as activated carbon and graphite are used in supercapacitor electrodes because of their high surface area, excellent electrical conductivity, and stability [25].

This difference in intrinsic conductivity was expected to influence the overall electrical behavior of the composite hydrogels, especially under mechanical stress or postdrying conditions.

### 2.2. Results of Hydrogel Preparation and Drying Treatments

The integration of carbonaceous materials into alginate-based hydrogels aims to enhance their electrical conductivity, mechanical integrity, and suitability for 3D printing. Selecting the appropriate formulation parameters, particularly alginate concentration and moisture content, is essential for achieving reproducible and functional constructs suitable for sensor applications.

**Figure 1:**
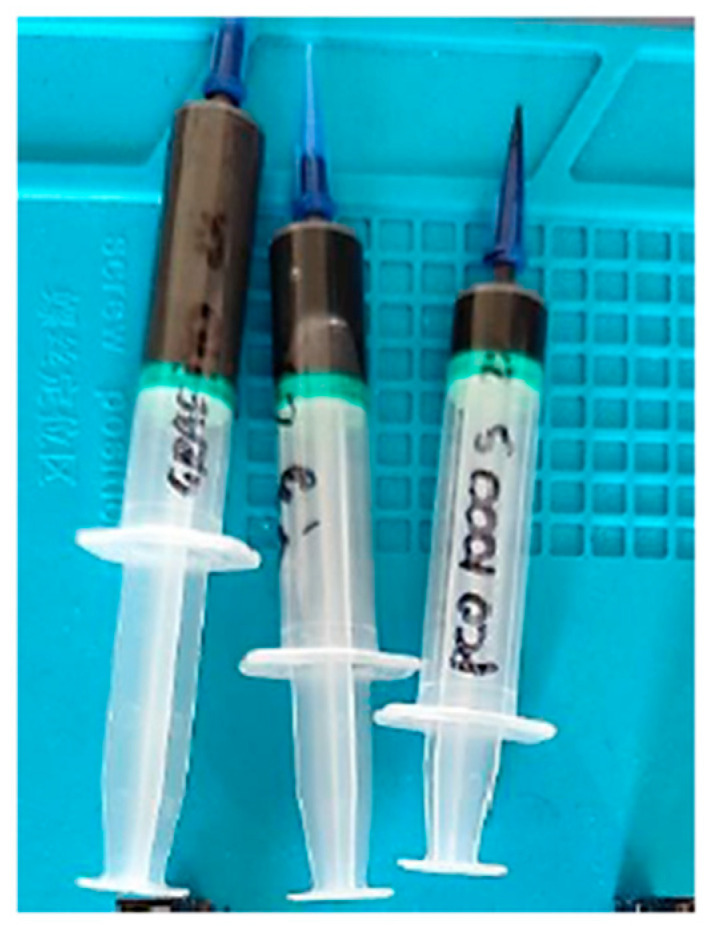
Preparation of hydrogels with carbonaceous elements.

Alginate concentrations of 1%, 1.5%, and 2% (*w*/*v*) were tested in combination with 8% (*w*/*w*) carbonaceous additives, including PCO1000C (activated carbon), Vulcan V3 (carbon black), and natural graphite. The hydrogel preparation process is illustrated in Figure 1, and the nomenclature used for the different formulations according to their drying treatment is summarized in Table 2.

Increasing alginate concentration typically results in a denser polymeric network, which may reduce porosity and ionic transport but improves mechanical strength and print fidelity. The 2% alginate formulation demonstrated the best balance between printability and structural robustness. These findings are in line with previous studies that report alginate concentrations ranging from 1–5% for biomedical uses [22], 0.1–1% for food industry applications [26], and up to 10% for industrial-grade hydrogels [27].

Moreover, ionic crosslinking also contributes to the final stiffness of the hydrogels. Calcium ions, for example, can create rigid structures even at relatively low alginate concentrations (1–3%), while other divalent cations such as magnesium may require higher polymer contents to achieve similar mechanical properties [28].

In parallel, drying strategies were assessed as a critical factor influencing the flow behavior and functional performance of the hydrogels. Three conditions were tested: oven drying at 50 °C, drying in open containers at room temperature, and drying in containers with perforated closures. Proper moisture control proved essential for modulating viscosity, mechanical resistance, and the formation of conductive pathways, especially relevant for applications involving extrusion-based printing and sensor response.

By systematically evaluating the interaction between alginate content, carbon phase, and drying conditions, a rational formulation was identified that supports extrusion, maintains mechanical stability, and enhances conductivity, as further discussed in the following sections.

#### Effect of Drying Conditions on Hydrogel Properties

The drying method significantly influenced the final properties of the carbon-loaded hydrogels, particularly with respect to moisture retention, printability, and structural stability. The percentage of water loss measured under different drying conditions is shown in Table 3.

Oven drying at 50 °C was the most effective strategy, yielding the highest water loss in all formulations. This condition promotes uniform and accelerated moisture removal, which favors the formation of a denser matrix and facilitates conductive pathway restoration. In contrast, drying in perforated containers resulted in lower water loss, likely due to slower evaporation and partial moisture retention, while open-container drying showed intermediate behavior.

Among the different formulations, Vulcan V3-based hydrogels exhibited the highest dehydration range across all conditions (86.37–92.05%), indicating a network more prone to moisture loss. Graphite-based hydrogels showed greater stability (84.00–85.90%), suggesting lower permeability and more consistent water retention. PCO1000C formulations displayed the greatest variability (85.68–94.90%), reflecting a strong dependence on the drying condition, likely due to its porous microstructure and surface chemistry.

These differences confirm that both the type of carbon additive and the drying method play key roles in defining the hydrogel’s functional characteristics. As reported in previous studies [29,30], drying control is essential for balancing print fidelity, mechanical strength, and electrical performance in conductive hydrogel systems.

### 2.3. Results of the Characterization of Hydrogel–Carbon Composites

The characterization of a hydrogel mixture with carbonaceous materials is a fundamental step in understanding its structure, properties and possible applications. This characterization allowed evaluating the effectiveness of the incorporation of carbonaceous materials in the hydrogel, as well as determining the final properties of the composite material.

#### 2.3.1. Results of Electrical Conductivity Tests

**Figure 2:**
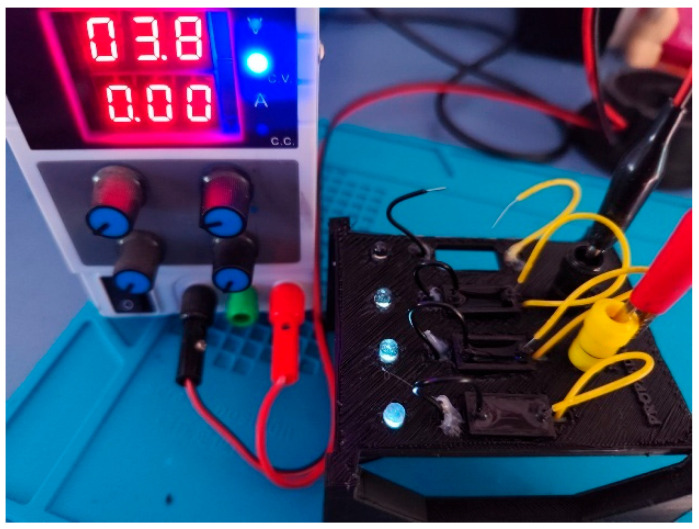
Device designed to quickly validate the electrical conductivity of hydrogels.

Following the preparation of the hydrogel formulations, an initial qualitative screening of electrical conductivity was performed in the liquid state to verify their potential suitability for sensor applications. Each hydrogel sample was placed inside a cuvette integrated into a custom-designed testing device (Figure 2), which applied a low-voltage electric potential across the sample. The successful activation of an LED indicated that the hydrogel exhibited sufficient conductivity to complete the circuit.

All tested hydrogel formulations, regardless of the carbonaceous additive used, successfully conducted electricity in their hydrated state, demonstrating baseline electrical functionality under standard conditions.

To evaluate their performance under realistic postprinting conditions, the electrical conductivity of each hydrogel was further assessed after a 48 h drying period, simulating the state of the material following extrusion and initial setting. Conductivity was measured under incremental mechanical pressure, reflecting practical use conditions in pressure-sensitive or contact-based sensors.

The results showed that conductivity improved notably with increased drying intensity, especially after oven drying at 50 °C, where the electrical performance approached that of the pristine carbonaceous fillers.

This behavior reflects the dual role of water in the hydrogel matrix. On one hand, it facilitates ion transport by solvating ionic species and contributes to the flexibility of the polymer network; on the other, it impedes efficient electron transfer between conductive particles by increasing the interparticle distance and preventing the formation of continuous percolation pathways. As previously reported by Su et al. (2024) [31], hydrated hydrogels rely primarily on ionic conductivity, and only upon dehydration does the electron-conductive mechanism emerge, driven by particle contact and matrix contraction.

Part of the applied stress is dissipated through polymer chain relaxation and water-mediated flow, while in drier hydrogels, the matrix exhibits more elastic behavior, directly transmitting compressive forces to the conductive phase without significant polymer rearrangement. This elastic response has been previously reported in dual-mode conductive hydrogels, where water loss leads to a solid-like state that promotes stress transfer through the polymer network rather than deformation [31].

Conversely, in drier samples, the polymer matrix becomes mechanically stiffer, reducing internal reorganization and allowing pressure to compact the carbon particles more effectively. This leads to decreased interparticle distances and increased formation of direct conductive bridges, promoting enhanced electron hopping and tunneling mechanisms. The observed linear conductivity gain with pressure in these samples reflected dominant percolation by mechanical densification rather than ionic contribution.

Therefore, water content critically modulates the interplay between polymer mobility and particle contact, determining whether conductivity arises predominantly from ionic mobility, matrix reconfiguration, or particle compaction under load.

In these formulations, the absence of residual water minimized matrix reorganization, and mechanical densification became the dominant mechanism enhancing conductivity.

**Figure 3:**
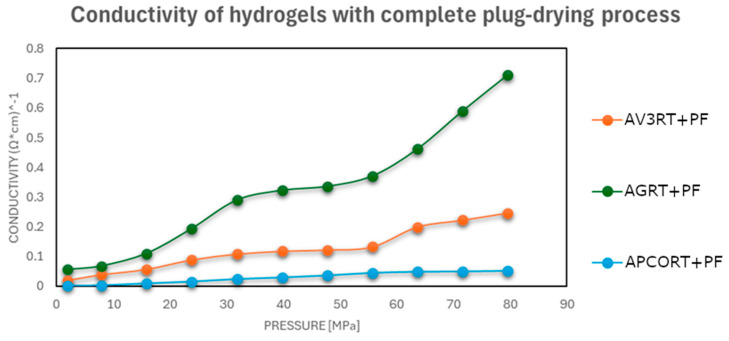
Electrical conductivities at different pressures of hydrogel–carbonaceous material mixtures after a drying process in a vessel with a perforated closure at room temperature.

**Figure 4:**
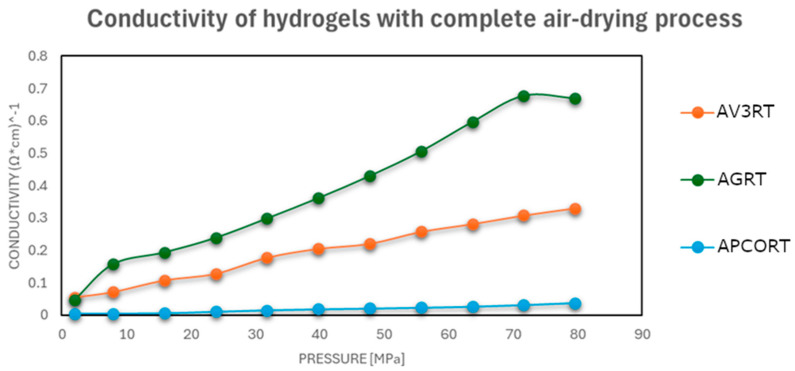
Electrical conductivities at different pressures of carbonaceous hydrogel–material mixtures after a drying process in an unsealed vessel at room temperature.

**Figure 5:**
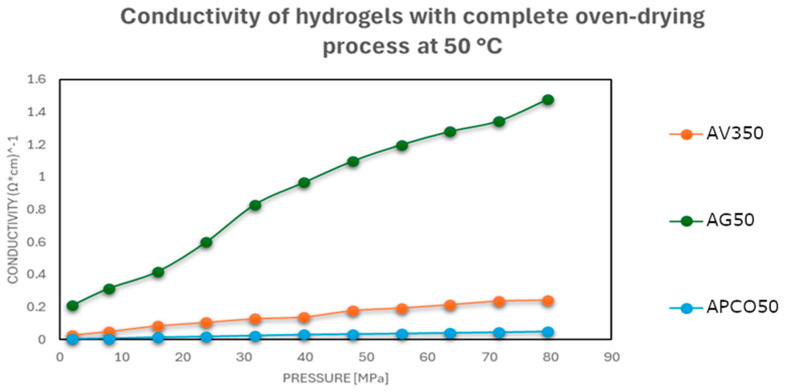
Electrical conductivities at different pressures of carbonaceous hydrogel–material mixtures after oven drying at 50 °C.

As shown in Figure 3, Figure 4 and Figure 5, a notable improvement in conductivity was observed in all formulations subjected to more intense drying, particularly oven drying at 50 °C. In these cases, the electrical conductivity values approached those of the original carbon materials, suggesting effective restoration of conductive pathways. In contrast, the samples dried in perforated or open vessels retained more water and exhibited lower conductivity, likely because of incomplete moisture removal and less favorable particle–particle interactions.

This pressure-dependent conductivity suggests a possible application in pressure- or strain-sensitive devices, where compaction of the material translates into measurable electrical changes.

In summary, water content plays a dual role in determining electrical conductivity: it affects both the packing density of carbonaceous particles and the structural flexibility of the polymer matrix under pressure [32]. Proper drying control is therefore essential to optimize electrical performance in conductive hydrogels designed for 3D-printed sensor applications.

#### 2.3.2. Results of Rheological Characterization

Preliminary rheological tests focused on stiffness, flow behavior, and viscosity revealed that alginate concentrations significantly influenced the extrudability and mechanical properties of the hydrogels. Formulations with alginate concentrations either below or above 2% (*w*/*v*) were found to be unsuitable for extrusion-based printing, as they exhibited either insufficient structural integrity or excessive viscosity that hindered flow through standard nozzles.

Based on these observations, the working concentration was set at 2% alginate, which provided a suitable balance between mechanical stability and flow behavior for 3D printing applications. The final compositions of the hydrogels containing different carbonaceous materials are summarized in Table 4.

**Figure 6:**
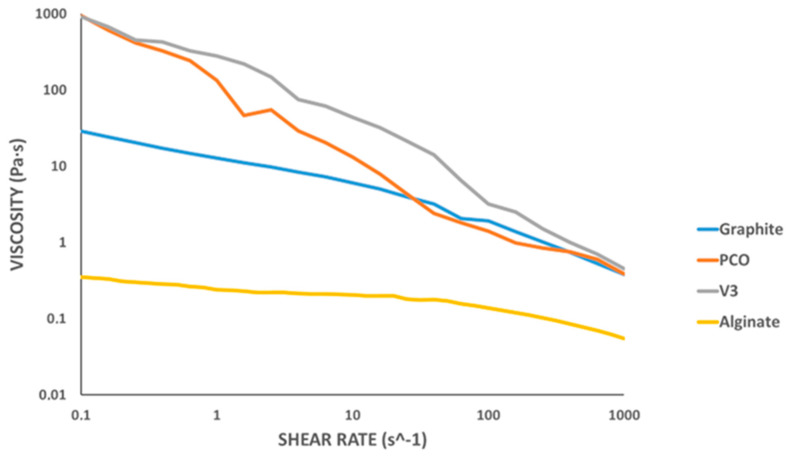
Shear rate versus shear viscosity rheological test at 50 °C.

The hydrogel formulations were prepared following the indicated procedure, and their rheological behavior was evaluated by measuring the shear viscosity as a function of shear rate at 50 °C (see Figure 6).

The hydrogel composed solely of alginate exhibited the lowest initial viscosity. Its viscosity decreased only slightly with increasing shear rate, in line with prior reports in the literature, and it showed the lowest initial viscosity [33,34].

The results clearly indicated pseudoplastic (shear-thinning) behavior for all formulations, characterized by a decrease in viscosity with increasing shear rate [35]. This behavior is typical of polymer-based hydrogels and is desirable for extrusion applications, as it facilitates flow under pressure while maintaining shape fidelity after deposition [36,37,38].

Among the tested samples, the formulations containing PCO1000C and Vulcan V3 exhibited higher initial viscosities, which may lead to increased flow resistance through the nozzle. It is important to note that extrusion performance is influenced not only by viscosity but by applied pressure and nozzle geometry. These factors must be considered when selecting materials for bioprinting processes.

In comparison with previous studies on alginate-based hydrogels reinforced with conductive additives, our formulations exhibited similar pseudoplastic behavior, yet with significant differences in initial viscosity and flow resistance depending on the nature of the incorporated carbonaceous additive. For instance, Serafin et al. [15] reported formulations containing carbon nanofibers (CNFs) with very high initial viscosities, which could lead to challenges during the printing process, including filament breakage and low geometric fidelity. In contrast, the formulations shown in Figure 6 maintained a pseudoplastic rheological profile without reaching such high viscosity levels. Notably, the graphite-based hydrogel exhibited the lowest initial viscosity among the tested samples, resulting in a lower extrusion pressure requirement and greater stability during the printing process. Moreover, unlike other approaches that require complex chemical modifications or copolymerization steps, such as those involving PVA/PANI hydrogels [13], the formulations presented in Table 4 were prepared by simple physical blending of alginate and carbon-based materials, which facilitates their fabrication and may enhance their biocompatibility for biomedical applications.

#### 2.3.3. Determining Optimum Printing Pressure

Following the rheological analysis and the evaluation of available bioprinting nozzle geometries [39,40], tests were performed to determine the optimal extrusion pressure required for each hydrogel formulation [41].

The geometry of the nozzle plays a fundamental role in extrusion-based printing, as it affects not only the quality and resolution of the printed structure but the flow dynamics of the material. The nozzle influences material distribution, reduces the risk of clogging, improves interlayer adhesion, and determines the surface finish of the printed construct. To identify the most suitable configuration, various nozzle types were tested, including standard cylindrical, angled orifice, oval, and rectangular nozzles.

Among these, the standard G20 rectangular nozzle demonstrated the best performance when used with the alginate–graphite hydrogel. This configuration provided a good balance between resolution and extrusion force, while maintaining print stability. In contrast, the other hydrogels (containing PCO1000C or Vulcan V3) were not extrudable under standard conditions because of excessive viscosity and high resistance to flow.

**Figure 7:**
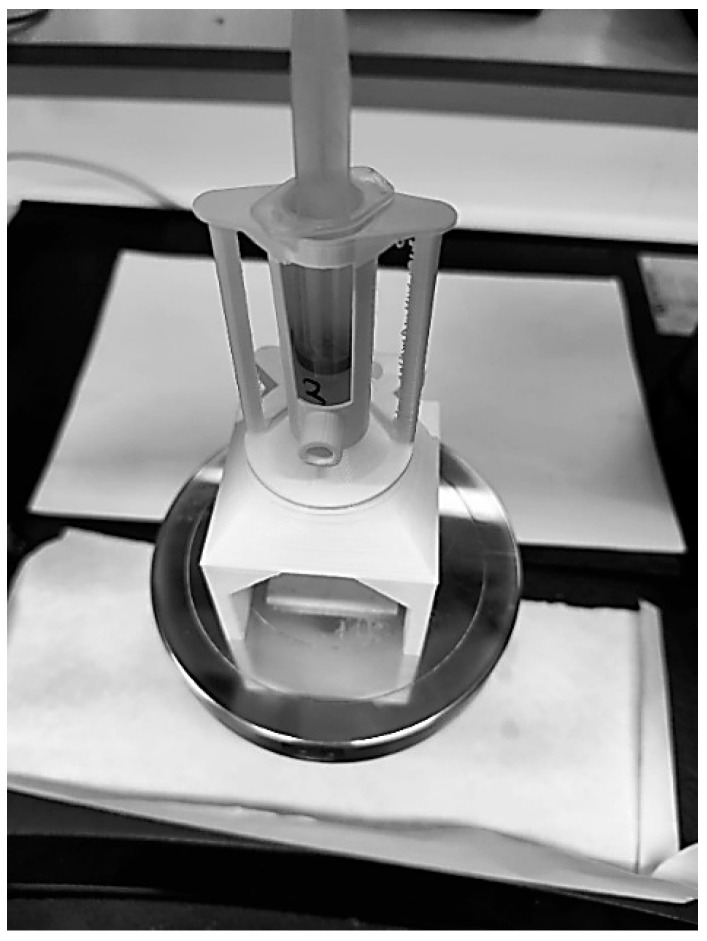
Measurement process to determine the pressure required to extrude hydrogel.

Once the nozzle type was selected, the extrusion force was quantified for each formulation using a custom-built testing system (Figure 7). The hydrogel was loaded into a syringe connected to the selected nozzle, and a controlled displacement force was applied by a load cell. The force required for the hydrogel to begin flowing was recorded and is presented in Table 5.

The relationship between applied force and extrusion behavior was analyzed by identifying the inflection point of the force–displacement curve, which corresponds to the minimum pressure needed to initiate continuous flow without overextrusion or deformation [42,43]. For the alginate–graphite hydrogel, this threshold was reached at approximately 0.008 kN, indicating that it possessed the necessary flow characteristics for precise and stable printing.

In contrast, the PCO1000C and Vulcan V3 formulations required forces close to 0.98 kN and 0.91 kN, respectively, values too high for practical bioprinting under standard conditions.

These results confirmed that only the graphite-based hydrogel formulation exhibited adequate extrudability under moderate pressure, making it the most suitable candidate for 3D-printed conductive hydrogel applications.

#### 2.3.4. Determination of 3D Printing Capability

Based on the previous extrusion and rheological analyses, the alginate–graphite hydrogel formulation was selected for experimental 3D printing trials because of its favorable printability, moderate viscosity, and stable flow behavior.

The ability to accurately print hydrogel-based structures with defined geometry is essential for applications in flexible sensors and other soft electronic platforms. To evaluate this capability, test cylindrical structures were fabricated using a modified 3D press for hydrogel extrusion, operating with the parameters listed in Table 6.

The hydrogel formulations were extruded prior to CaCl_2_ crosslinking. Because of the low aspect ratio and planar geometry of the printed discs, structural collapse was not a concern, and the native viscosity of the ink was sufficient to preserve the shape during deposition. Final stabilization was achieved by CaCl_2_ application after printing.

**Figure 8:**
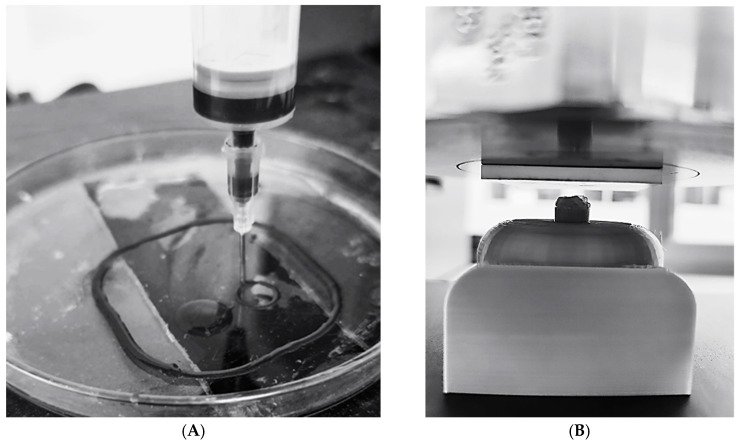
(**A**) Three-dimensional printing of hydrogel with graphene in a cylindrical shape; (**B**) process of measuring the capacity of the cylindrical hydrogel to resist loads.

As shown in Figure 8A, the hydrogel was successfully extruded and printed into self-supporting cylindrical structures, indicating good shape fidelity and adequate material cohesion. To further evaluate the mechanical performance of the printed hydrogels, compressive tests were carried out on the printed cylinders before and after ionic crosslinking with calcium chloride.

Compression tests were conducted at 28%, 57%, and 75% strain, and both the force required for deformation and the compressive strength after crosslinking were recorded (Table 7). The experimental setup is illustrated in Figure 8B.

These results demonstrate that the printed alginate–graphite hydrogel structures exhibited a clear increase in mechanical resistance with higher compressive strain and that ionic crosslinking significantly enhanced their load-bearing capacity. After crosslinking, the resistance to compression increased from 0.056 N at 28% strain to 0.99 N at 75% strain (an almost 18-fold improvement), highlighting the hydrogel’s ability to maintain structural cohesion under mechanical stress.

In practical terms, even a lightweight printed hydrogel structure (0.35 g) was able to withstand more than 150 times its own weight (0.056 N) at just 28% deformation. This indicates that the hydrogel is not only printable and self-supporting but mechanically robust enough to support additional printed layers, which is essential for multilayer fabrication in 3D printing.

The combination of dimensional fidelity, compressive strength, and postprinting mechanical enhancement via crosslinking confirms the potential of this formulation for wearable and implantable soft sensors, particularly in applications requiring tactile response, pressure sensitivity, or structural resilience in dynamic environments.

#### 2.3.5. Results of the Cytotoxicity Assay

**Figure 9:**
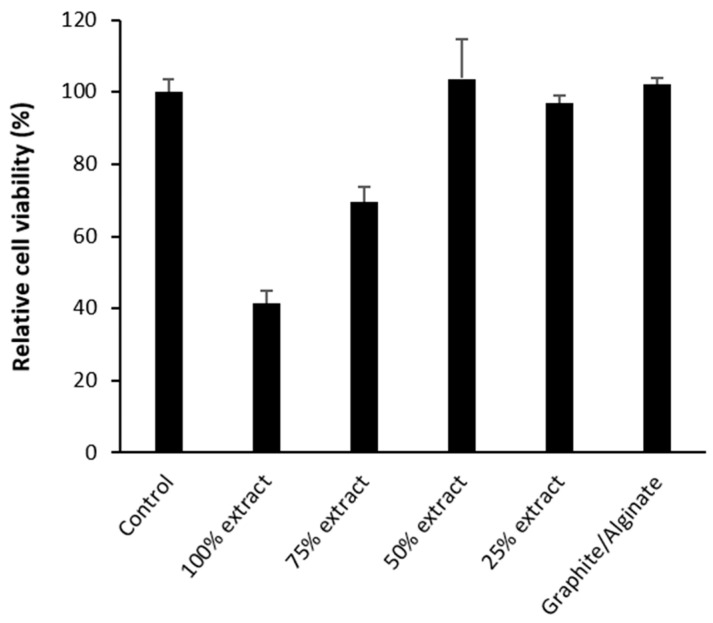
Cytotoxicity results by MTS assay of graphite extract at different concentrations and alginate–graphite printed discs. MCF7 cells were in contact with each element for 24 h. Values correspond to means ± SD of assays performed in triplicate.

Cell viability in the presence of graphite extracts at different concentrations, as well as after direct contact with printed alginate–graphite hydrogel discs, was assessed by MTS assay. Figure 9 shows that exposure of cells to graphite extract showed a concentration-dependent trend, with minimum cell viability observed with 100% extract and a gradual increase in viability with decreasing extract ratio. In particular, mean viability values increased progressively in the groups treated with 75%, 50%, and 25% extract, reaching levels comparable to the control at 50% dilution. This trend suggests a negative dose–response relationship between extract concentration and cell survival, coinciding with results obtained in previous studies [44]. In addition, other studies have demonstrated the toxicity of compounds such as nanographite and nanoparticles [45], which, because of its size (10–100 nm), could pass through the 0.22 um filter, exerting a cytotoxic effect on the cells in contact with the extract [46].

At 50% dilution and above, no significant differences were observed with respect to the negative control, indicating that the toxicity induced by free graphite was substantially reduced below this concentration.

In contrast, direct contact with the alginate–graphite hydrogel discs did not induce significant alterations in cell viability compared with the control, which was around 100%. This behavior can be attributed to the high adsorption capacity of the graphite powder, which can sequester components present in the culture medium, such as phenol red [47], vitamins [48], amino acids [49] o factors present in fetal bovine serum [50].

Furthermore, studies have also indicated that graphite was able to adsorb both synthetic and organic dyes [51], as well as industrial effluents containing compounds such as phenol [52].

The loss of color of the medium observed after incubation with graphite reinforces this hypothesis and suggests an alteration in the composition of the culture medium that compromises the chemical balance of the cellular environment, which could have contributed to the decrease in viability.

In contrast, no change in the staining of the culture medium and no decrease in cell viability were observed when the alginate–graphite discs were placed in contact with the cell culture. Other studies have shown that coating carbonaceous compounds with sodium alginate reduced toxicity and improved compatibility [53], This may suggest that when graphite is immobilized within an alginate matrix, its interaction with the medium is more limited, and therefore, its cytotoxic effects are less severe.

This finding is in line with previous work highlighting the importance of the immobilization of nanomaterials or carbonaceous particles in polymeric matrices to preserve biocompatibility [54,55]. Thus, the immobilization of graphite in a hydrogel network could act as a physical and chemical barrier, preventing both direct contact with the cells and the alteration of the components of the medium.

These results reinforce the potential of alginate–graphite hydrogel as a biocompatible support for sensor applications and suggest that the presentation form of graphite is a critical factor for its biosafety.

## 3. Conclusions

This work presents the development and characterization of a new family of printable and conductive hydrogels based on alginate and carbonaceous materials, with potential applications in the fabrication of flexible and biocompatible sensors.

The drying method significantly influences the electrical conductivity of the composites. Oven drying at 50 °C was the most effective strategy for restoring conductive pathways by facilitating interparticle contact and reducing water retention.

Among the tested formulations, only the alginate–graphite hydrogel demonstrated suitable rheological properties, extrudability, and mechanical stability for 3D printing under practical conditions. The printed constructs showed good shape fidelity and compressive resistance, especially after ionic crosslinking.

Finally, cytotoxicity assays confirmed the biocompatibility of the printed alginate–graphite hydrogel, supporting its safe integration into biomedical environments. These results highlight the suitability of this formulation as a promising material for use in wearable sensors, implantable devices, and other soft bioelectronic applications.

In future developments, this alginate and graphite hydrogel could be functionalized for use in nonenzymatic electrochemical biosensors. Given the intrinsic conductivity of the material and the ease of immobilizing functional groups within the alginate matrix, molecules could be incorporated to improve selectivity and sensitivity. These strategies would enable the design of low-cost, single-use biosensor patches, and their simplicity, biocompatibility, and ease of printing make them a promising platform for prototyping portable diagnostic tools, especially in resource-limited settings.

## 4. Materials and Methods

### 4.1. Starting Materials

The following materials were used in this research work:

➢ Commercial charcoal PCO1000C, supplied by GalaQuim (Madrid, Spain).
➢ Vulcan 3 carbon black, supplied by Cabot Corporation (Madrird, Spain).
➢ Natural graphite (battery grade, <45 μm particle size, purity of ≥99.99% trace metals basis) supplied by Merck (Madrid, Spain).
➢ Sodium alginate, supplied by Sigma Aldrich (Madrid, Spain).
➢ Calcium chloride, supplied by Sigma Aldrich.

Carbonaceous materials were selected to evaluate the influence of carbon allotropes on the results.

### 4.2. Electrical Characterisation

The measurement of the electrical conductivity in different pressure ranges was carried out with equipment patented by us (Patent U202032279), which was coupled to an INSTRON 5565 universal testing machine [56] that allowed different pressure ranges to be applied. This equipment not only made it possible to determine the electrical conductivity but to obtain a trend line that facilitated the deduction of the behavior of the material under different pressure magnitudes [57]. Through this measurement method, it is possible to use the trend line to extrapolate electrical conductivity values that the material could present under other pressure conditions or with different amounts of material introduced.

### 4.3. Preparation of the Hydrogel Mixture with Carbonaceous Material

Hydrogels, because of their high water-absorption capacity and biocompatibility, have found numerous applications in fields such as biomedicine and tissue engineering. The incorporation of carbonaceous materials into these systems offers the possibility of developing composite materials with improved electrical and mechanical properties.

In this study, a composite hydrogel was prepared to obtain a highly conductive material with good biocompatibility. For this purpose, a hydrogel with sodium alginate as a matrix and activated carbon PCO1000C, Vulcan V3 carbon black, and graphite as carbonaceous materials were used.

#### 4.3.1. Preparation of Hydrogels

Alginate hydrogels with different concentrations (1%, 1.5%, and 2%) were prepared by dissolving corresponding amounts of alginate in 180 mL of hot water. Details of the preparation are given in Table 8.

To each hydrogel, 3.4 g of either PCO1000C, graphite, or V3 was added. Each mixture was labeled according to the nomenclature shown in Table 8.

The mixtures with their corresponding carbon additives were stored in a refrigerator for 24 h.

#### 4.3.2. Drying Process

Three samples of each mixture prepared were evaluated to analyze the influence of the drying process:

➢ Drying in a container with a perforated closure (PF) at room temperature (22 °C).
➢ Drying in an unsealed container at room temperature (RT) (22 °C).
➢ Drying in an unsealed container in an oven at 50 °C.

The weight loss of each sample was measured every 30 min for the first 2 h, and then daily for the next 48 h to track the moisture loss and its influence on the hydrogel’s final properties.

Water content plays a critical role in determining the printability, mechanical stability, and electrical behavior of carbon-loaded hydrogels, especially in extrusion-based 3D printing. Excessive moisture tends to reduce viscosity, improving flow but compromising structural fidelity, while low water content increases viscosity and dimensional stability at the expense of extrudability. Furthermore, hydration levels affect the elastic response and compressive strength of the printed structures. Understanding and controlling the drying process is therefore essential to achieve reproducible and functional properties in the final hydrogel formulations.

### 4.4. Characterization of Hydrogel Mixtures with Carbonaceous Materials

Hydrogels are widely used in biomedicine and tissue engineering because of their high-water retention capacity and excellent biocompatibility. Incorporating carbon-based fillers into these matrices can significantly enhance their electrical and mechanical properties, enabling their use in pressure sensors, soft electronics, and biosensing platforms.

In this study, composite hydrogels were formulated using sodium alginate as the biopolymeric matrix. To impart conductivity, three types of carbonaceous materials were incorporated: PCO1000C (activated carbon), Vulcan V3 (carbon black), and natural graphite. These materials were selected for their distinctive physicochemical profiles, including differences in surface area, particle size, and morphology, which have been previously characterized in the literature [58,59,60,61]. This allowed us to focus on the processing behavior and functional integration into the hydrogel system.

The hydrogel mixtures were prepared at varying alginate concentrations, and the influences of both the carbon additive and the drying strategy on performance were assessed. The specific preparation protocol and drying conditions are described in detail in following sections.

#### 4.4.1. Electrical Conductivity

The electrical conductivity of the previously prepared hydrogel samples was evaluated in order to identify the most suitable formulations for sensor-oriented applications.

**Figure 10:**
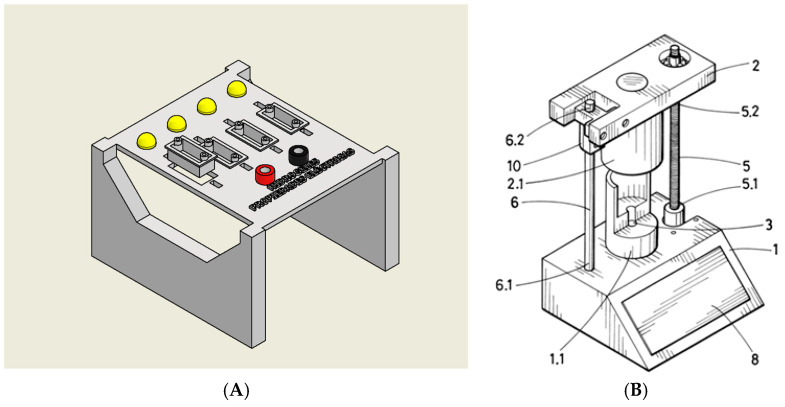
(**A**) The system consisted of a set of cuvettes connected to a device that applied a voltage via a power supply. If the hydrogel was conductive, the circuit was completed, and an LED was activated as a visual indicator of the conductivity. (**B**) Patent developed by the authors at the University of Extremadura (U202032279) [62].

As a first step, a rapid screening test was carried out to determine whether each hydrogel exhibited conductive behavior in its liquid state. For this purpose, each formulation was placed into a custom-designed testing chamber (Figure 10A) consisting of a small cuvette connected at one end to a power supply and at the other to a LED. The circuit closed only in the presence of a conductive hydrogel, thereby enabling a quick and intuitive confirmation of its electrical properties.

For a more detailed and quantitative assessment, conductivity was subsequently measured using a patented device (Patent U202032279) (Figure 10B). This system enabled the application of controlled compressive forces to the hydrogel samples while simultaneously measuring their electrical conductivity. By analyzing the conductivity response under varying pressure levels, it was possible to determine the formulation with the most favorable electromechanical performance for potential use in soft, flexible sensor systems.

#### 4.4.2. Rheological Characterization

Following the electrical conductivity analysis, the rheological properties of the hydrogel formulations were evaluated using a rheometer [62]. Viscosity, defined as the resistance to flow caused by internal friction, is closely related to the microscopic characteristics of the system, including particle size and polymer network structure. Studying the viscosity behavior under different shear rates provides insight into the flow dynamics of the hydrogels, their shear-thinning behavior, and their ability to be extruded through a nozzle, key factors for applications involving 3D printing or direct ink writing.

**Figure 11:**
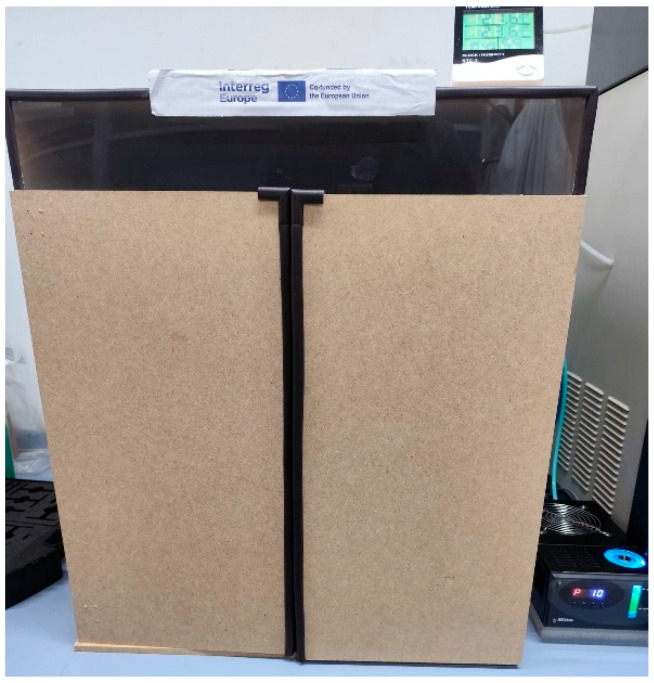
Room where the rheometer was located to monitor and control the temperature and humidity at which the tests were performed.

To ensure consistent and reliable measurements across all formulations, the rheometer was operated within a controlled environment. The device was placed on a platform equipped with real-time monitoring and regulation of temperature and ambient humidity, minimizing potential sources of experimental error. During the tests, the measurement chamber was maintained at a constant temperature of 24 °C and a relative humidity of 59%, thereby ensuring reproducibility and stability of the rheological data (Figure 11).

#### 4.4.3. Methodology for Determining Optimum Printing Pressure

To determine the optimum pressure conditions for printing, it was essential to assess both the geometry of the nozzle and the force required to extrude each hydrogel formulation. The shape and size of the nozzle play critical roles in extrusion-based 3D printing, as they directly influence the resolution, surface finish, and structural fidelity of the printed constructs.

**Figure 12:**
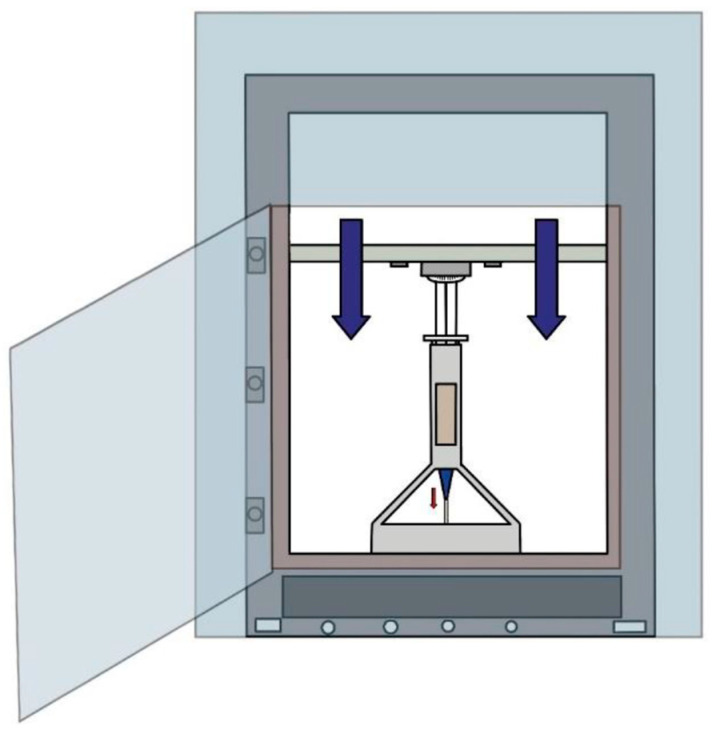
Equipment for determining the optimum printability pressure (authors’ patent pending).

The evaluation was carried out using a custom-designed device (Figure 12, patent pending) developed by the authors, which allowed precise control over environmental conditions, extrusion temperature, and displacement speed.

To assess the extrusion behavior, each hydrogel formulation was loaded into a syringe fitted with a selected nozzle. The syringe was mounted onto the custom equipment, and a range of movement speeds was programmed for the load cell. During the test, the load cell descended at a constant speed while continuously recording the force required to initiate flow through the nozzle, along with the temperature of the hydrogel and ambient chamber (Figure 12).

This methodology enabled the identification of the minimum force (and corresponding pressure) necessary for extrusion without deformation or interruption of the filament. Once the optimal parameters (extrusion pressure, temperature, and speed) were established, the samples were transferred to a commercial 3D printer modified for use with hydrogels and with the ability to apply heat and pressure in a controlled manner.

#### 4.4.4. Methodology for the Determination of 3D Printing Capability

To evaluate the ability of carbon-loaded hydrogels to reproduce predefined geometries, a cylindrical model was designed using Autodesk^®^ Inventor^®^ 2025. Based on the optimal extrusion parameters previously obtained (see Table 9), printing conditions (pressure, speed, and temperature) were configured in the open-source software UltiMaker Cura 5.8, which was used to generate the G-code for printing. The printing process was carried out using an Ender 3 3D printer modified for application with hydrogels. A pneumatic print head designed to accommodate syringes was incorporated, which included a heatable electrical resistor and an integrated thermistor for precise temperature generation and control. The printed elements were deposited in Petri dishes.

In addition to geometric fidelity, the mechanical stability of the printed hydrogels was evaluated under compression to determine their capacity to retain shape under load. For this purpose, cylindrical hydrogel specimens (formulated with different carbonaceous materials) were subjected to compressive strain tests using a universal testing machine (6800 Series Universal Testing Systems). A controlled, downward force was applied until reaching 28%, 57%, and 75% deformation of the sample’s original volume. The compressive force sustained by each hydrogel was recorded to quantify its mechanical integrity.

Given the known crosslinking ability of alginate, the effect of ionic crosslinking on the compressive strength was also investigated. Comparisons were made between uncrosslinked and crosslinked samples to assess how this parameter influences structural performance under mechanical stress. The samples were crosslinked with 50 mM calcium chloride [63].

#### 4.4.5. Cytotoxicity Assay

MCF-7 cells were seeded in a 96-well plate at a density of 5000 cells per well and left for 24 h to reach subconfluence in complete DMEM culture medium (supplemented with 10% FBS and 1% pen/strep; culture conditions: 37 °C, 5% CO_2_). Two different analyses were performed:

- Graphite powder (0.2 g/mL) was incubated in DMEM with 10% fetal bovine serum (FBS) and 1% penicillin/streptomycin for 24 h to obtain the extract, which was filtered and placed in contact with cell cultures at different concentrations for another 24 h.
- Hydrogel discs formulated with alginate and graphite, previously 3D printed, oven dried, and sterilized by ultraviolet radiation, were placed directly on previously attached cell cultures. After 24 h of incubation, discs were removed.

In both cases, cell viability was analyzed using the MTS assay (CellTiter 96^®^ AQueous One Solution, Promega), measuring absorbance at 490 nm with an iMark™ microplate reader. All assays were performed in triplicate (*n* = 3).

## Figures and Tables

**Table 1:** Electrical conductivity values at room temperature.

Samples	Pressure (MPa)	Conductivity (Ω·cm)^−1^
PCO1000C	48	1.11
Vulcan V3	48	2.21
Graphite	48	6.12
Sodium alginate	48	0.050

**Table 2:** Nomenclatures of samples with different drying treatments.

Samples	Nomenclature
Alginate + PCO1000C + RT.	APCORT
Alginate + PCO1000C (50 °C)+ RT.	APCO50RT
Alginate + PCO1000C+ RT. + Perforated Fastener	APCOTA+PF
Alginate + Vulcan V3+ RT.	AV3RT
Alginate + Vulcan V3 (50 °C) + RT.	AV350RT
Alginate + Vulcan V3 + RT. + Perforated Fastener	AV3TA+PF
Alginate + Graphite + RT.	AGRT
Alginate + Graphite (50 °C) + RT.	AG50RT
Alginate + Graphite + RT. + Perforated Fastener	AGTA+PF

**Table 3:** Percentage weight losses of carbonaceous hydrogel–material mixtures.

Drying/Sample	Hydrogel–Vulcan V3	Hydrogel–Graphite	Hydrogel–PCO1000C
Drying with perforated closure	89.72	84.00	85.68
Drying without sealing	86.37	84.15	85.96
Oven drying at 50 °C	92.05	85.90	94.90

**Table 4:** Composition of hydrogel mixtures with carbonaceous materials.

Carbonaceous Material	Alginate	Water
PCO1000C (8%)	2%	90
Vulcan V3 (8%)	2%	90
Graphite (8%)	2%	90

**Table 5:** Results of the measurement process to determine the pressure required to extrude the hydrogels.

HYDROGEL	Force [KN]
Hydrogel with Graphite	0.008
Hydrogel with PCO1000C	1.45
Hydrogel with Vulcan V3	1.18

**Table 6:** Parameters entered in the 3D bioprinter for printing.

Print Parameters	Value
Temperature	50 [°C]
Pressure	70 [kPa]
Speed	40 [mm/s]

**Table 7:** Hydrogel resistance to compressive loads.

Description	Weight [g]	Compression [%]	Resistance [N] to Being Compressed	Compressive Strength [N] After Crosslinking
Hydrogel with graphite	0.35	28	0.032	0.056
		57	0.088	0.28
		75	0.25	0.99

**Table 8:** Preparation of hydrogel mixtures with carbonaceous materials.

Material	Volume H_2_O [millilitres]	Concentration Solution	Volume Solution	Material 2	Nomenclature
1.8 g alginate	180	1%	20 mL to 1%	3.4 g PCO1000C	A1PCO
1.8 g alginate	180	1%	20 mL to 1%	3.4 g graphite	A1G
1.8 g alginate	180	1%	20 mL to 1%	3.4 g V3	A1 V3
2.7 g alginate	180	1.5%	20 mL to 1.5%	3.4 g PCO1000C	A1.5PCO
2.7 g alginate	180	1.5%	20 mL to 1.5%	3.4 g graphite	A1.5G
2.7 g alginate	180	1.5%	20 mL to 1.5%	3.4 g V3	A1.5V3
3.6 g alginate	180	2%	20 mL to 2%	3.4 g PCO1000C	A2PCO
3.6 g alginate	180	2%	20 mL to 2%	3.4 g graphite	A2G
3.6 g alginate	180	2%	20 mL to 2%	3.4 g V3	A2V3

**Table 9:** Parameters of the 3D printed and parameters used for the printing process.

**Geometry**	**Value**
Diameter	9 [mm]
Height	3 [mm]
**Print Parameters**	**Value**
Temperature	50 [°C]
Pressure	Determined for each hydrogel
Speed	40 [mm/s]

## Data Availability

The authors confirm that the data supporting the findings of this study are available within the article.
